# Mutations in Ehrlichia chaffeensis Genes ECH_0660 and ECH_0665 Cause Transcriptional Changes in Response to Zinc or Iron Limitation

**DOI:** 10.1128/JB.00027-21

**Published:** 2021-06-08

**Authors:** Ascención Torres-Escobar, María D. Juárez-Rodríguez, Roman R. Ganta

**Affiliations:** a Center of Excellence for Vector-Borne Diseases, Department of Diagnostic Medicine/Pathobiology, College of Veterinary Medicine, Kansas State University, Manhattan, Kansas, USA; Brigham and Women's Hospital/Harvard Medical School

**Keywords:** *Rickettsiales*, *Anaplasmataceae* pathogens, tick-borne diseases, metal ion deficiency, *Ehrlichia chaffeensis*, iron, metal ions, zinc

## Abstract

Ehrlichia chaffeensis causes human monocytic ehrlichiosis by replicating within phagosomes of monocytes/macrophages. A function disruption mutation within the pathogen’s ECH_0660 gene, which encodes a phage head-to-tail connector protein, resulted in the rapid clearance of the pathogen *in vivo*, while aiding in induction of sufficient immunity in a host to protect against wild-type infection challenge. In this study, we describe the characterization of a cluster of seven genes spanning from ECH_0659 to ECH_0665, which contained four genes encoding bacterial phage proteins, including the ECH_0660 gene. Assessment of the promoter region upstream of the first gene of the seven genes (ECH_0659) in Escherichia coli demonstrated transcriptional enhancement under zinc and iron starvation conditions. Furthermore, transcription of the seven genes was significantly higher under zinc and iron starvation conditions for E. chaffeensis carrying a mutation in the ECH_0660 gene compared to the wild-type pathogen. In contrast, for the ECH_0665 gene mutant with the function disruption, transcription from the genes was mostly similar to that of the wild type or was moderately downregulated. Recently, we reported that this mutation caused a minimal impact on the pathogen’s *in vivo* growth, as it persisted similarly to the wild type. The current study is the first to describe how zinc and iron contribute to *E. chaffeensis* biology. Specifically, we demonstrated that the functional disruption in the gene encoding the phage head-to-tail connector protein in *E. chaffeensis* results in the enhanced transcription of seven genes, including those encoding phage proteins, under zinc and iron limitation.

**IMPORTANCE** Ehrlichia chaffeensis, a tick-transmitted bacterium, causes human monocytic ehrlichiosis by replicating within phagosomes of monocytes/macrophages. A function disruption mutation within the pathogen’s gene encoding a phage head-to-tail connector protein resulted in the rapid clearance of the pathogen *in vivo*, while aiding in induction of sufficient immunity in a host to protect against wild-type infection challenge. In the current study, we investigated if the functional disruption in the phage head-to-tail connector protein gene caused transcriptional changes resulting from metal ion limitations. This is the first study describing how zinc and iron may contribute to *E. chaffeensis* replication.

## INTRODUCTION

The obligate intracellular rickettsial pathogen Ehrlichia chaffeensis grows within the phagosomes of monocytes/macrophages and causes human monocytic ehrlichiosis (HME), a potentially life-threatening zoonotic disease (1, 2). This pathogen is transmitted primarily from an infected Amblyomma americanum tick (3, 4). Common signs and symptoms of HME range from influenza-like symptoms to a life-threatening disease (5). E. chaffeensis also causes a similar disease in dogs, in addition to infecting white-tailed deer and coyotes (6). Several other obligate tick-transmitted Anaplasmataceae family pathogens, including Ehrlichia ewingii, Ehrlichia canis, Ehrlichia muris subspecies *eauclairensis*, and Anaplasma phagocytophilium, are also known to cause diseases in people (7–9). These pathogens have reduced genomes and are also evolved for intracellular survival within arthropod hosts and cause persistent infections in vertebrate hosts (6). *E. chaffeensis* replication within phagosomes inhibits phagosome-lysosome fusion, and the pathogen evades the host immune responses by several mechanisms (10–18). *Ehrlichia* species and other related Anaplasmataceae pathogens depend on the host for nutritional sources such as metabolites and metal ions for survival (6, 19).

While iron and zinc are two essential metal ions, neither of them is readily accessible within a host for an intracellular pathogen (20). Zinc and iron are also known to play essential roles in host innate and adaptive immune functions (21, 22). During pathogen infection, nutritional immunity can be triggered that involves the host-imposed metal ion deprivation or poisoning to counter infection progression (23, 24). Iron can catalyze the formation of reactive oxygen species (ROS) during macrophage-based bacterial killing by damaging bacterial membranes, proteins, and DNA (25). Bacterial pathogens also employ proficient ways to regulate the acquisition of the metal ions to both support nutritional requirements and to evade metal ion-mediated host response (24). Previous studies reported that E. chaffeensis-containing phagosomes and, similarly, Mycobacterium tuberculosis-containing phagosomes in human monocytes accumulate transferrin receptors (26, 27). *E. chaffeensis* inclusions are early endosomes, which selectively accumulate transferrin receptors, possibly facilitating the acquisition of iron by the pathogen from the host cell cytoplasm. A recent study described upregulation of iron-regulated genes under iron starvation conditions in a related bacterium, Ehrlichia ruminantium, by a predicted master regulatory protein, ErxR (28).

Simplification and assimilation of bacteriophage genomes into bacterial chromosomes is well documented (29). Such a process, leading to residual phage genomes being integrated into bacterial chromosomes, serves as a secretion system to support bacterial growth or provide enzymes that benefit the bacteria by helping them to adapt to adverse environments (30–32). The bacteriophage-derived secretion systems in bacteria are used for multiple functions, including the translocation of effector proteins and metal ion transporters (30, 33). Rickettsial pathogens, including *E. chaffeensis*, contain only two known secretion systems, the type I secretion system (T1SS) and the type IV secretion system (T4SS) (6). We recently discovered that a function disruption mutation within a *E. chaffeensis* gene (ECH_0660) encoding a phage head-to-tail connector protein causes the bacterial attenuation and leading to its rapid clearance from vertebrate hosts (34, 35).

In the current study, we assessed how the ECH_0660 mutation contributes to *E. chaffeensis* attenuation by performing molecular characterization of the seven genes spanning from ECH_0659 to ECH_0665, as four genes from this cluster encode phage-related proteins, including ECH_0660. We described how deprivation of zinc and iron impacts gene expression from the gene cluster in wild-type *E. chaffeensis* and mutants of the pathogen with gene function disruptions in two of the seven genes, ECH_0660 and ECH_0665.

## RESULTS

### Analysis of *E. chaffeensis* DNA sequence surrounding the ECH_0660 gene.

A genomic segment spanning *E. chaffeensis* genes ECH_0659 to ECH_0665 (seven genes) was evaluated for its relationship with the ECH_0660 gene encoding the phage head-to-tail connector protein in which we reported a disruption mutation impacting *in vivo* growth (34) (see Fig. S1 in the supplemental material). Intergenic spacer sequences are absent or ranged in size from 43 to 141 bp long in some of the seven genes assessed. The intergenic spacer regions are absent between the coding regions of ECH_0660, ECH_0661, and ECH_0662. Furthermore, the protein coding sequences for these three genes are overlapped by a few nucleotides, namely, four nucleotides between ECH_0660 and ECH_0661 and 23 nucleotides between ECH_0661 and ECH_0662. ECH_0659 encodes a hypothetical protein, while ECH0661, ECH_0662, and ECH_0665 are similar to ECH_0660 in encoding predicted bacteriophage proteins, phage major tail protein, phage stopper protein, and phage terminase large subunit protein, respectively. Structural prediction analysis revealed a greater degree of structural homology for the four predicted phage proteins of *E. chaffeensis* with previously documented structural phage proteins and a protein required for phage DNA packaging (see Fig. S2 in the supplemental material) (36). ECH_0663 and ECH_0664 genes are paralogs encoding hypothetical proteins sharing 34.5% homology. To determine if the seven genes are conserved in other related Anaplasmataceae family bacteria, genomes of seven organisms belong to the genera Anaplasma, Ehrlichia, Neorickettsia, and Wolbachia were compared (see Fig. S3 in the supplemental material). Homologs for all seven genes were identified in other *Ehrlichia* species. Anaplasma phagocytophilum lacks only an ECH_0663 homolog (one of the two paralogs), while both ECH_0663 and ECH_0664 homologs are absent in Anaplasma marginale and *Wolbachia*. Neorickettsia sennetsu contains only one of the seven genes.

### Promoter segment upstream of the first gene of the seven genes responds to the metal ion restriction.

**FIG 1:**
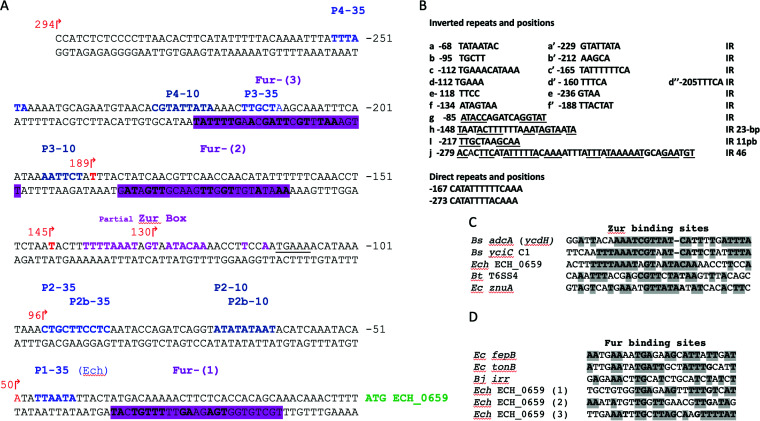
The putative promoter region upstream of the ECH_0659 coding region contains DNA binding motifs that likely contribute to metal ion regulation and transcription. (A) Putative promoter region upstream of the ECH_0659 coding region. Regions that resemble the consensus for the Fur-binding site are indicated by magenta shading and in boldface type and are labeled Fur-(1), Fur-(2), and Fur-(3). The region that resembles the consensus for the Zur-binding site is indicated in boldface type in magenta color and labeled “partial Zur box.” Predicted −35 and −10 sequences are shown in blue and cyan-blue text, respectively, and are labeled P1-35, P2-35, P2b-35, P3-35, P4-35, P2-10, P2b-10, P3-10, and P4-10. (B) Inverted repeat sequences that are separated are a-a′, b, b′, c-c′, d-d′-d″, e-e′ and f-f′; inverted repeats that are located at close proximity are identified with underlines and listed as h, i, and j. Direct repeats are shown after the inverted repeats. (C and D) Zur (C) and Fur (D) partial consensus binding site sequences from other Gram-positive and Gram-negative bacteria are indicated by gray shading. *Bt*, Burkholderia thailandensis; *Bs*, Bacillus subtilis; *Bj*, Bradyrhizobium japonicum; *Ec*, E. coli; *Ech*, *E. chaffeensis*.

**FIG 2:**
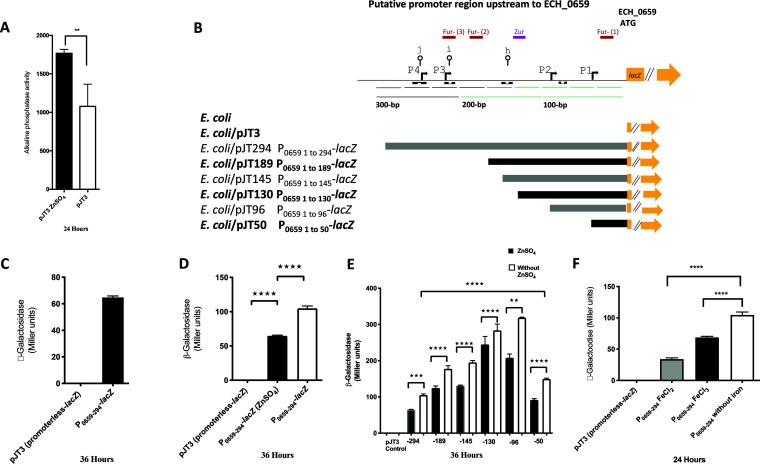
A promoter region upstream of ECH_0659 assessed in an Escherichia coli surrogate system using zinc- and iron-sufficient or depletion conditions. (A) Endogenous alkaline phosphatase activity (PhoA) of the E. coli XL1-Blue MRF′ strain harboring the plasmid vector pJT3. Bacterial cells were grown in chemically defined medium (Tris-glucose) with zinc or phosphate restriction or supplemented with 15 μM ZnSO_4_ or 64 mM buffer phosphate. For iron response, bacterial cells were grown in Tris-glucose medium, iron depleted or supplemented with 100 μM FeCl_2_ or 100 μM FeCl_3_, as described in Materials and Methods. (B) Schematic diagram of the ECH_0659-promoter region and transcriptional fusion constructed pJT294, pJT189, pJT145, pJT130, pJT96, pJT50, and the control pJT3 plasmid, showing the putative binding regions for Zur and Fur (colored boxes), the −10 and −35 promoter elements (bent arrows), and the secondary structures (lines with open circles [j, i, and h]). Inverted repeat sequences are indicated with black boxes. The numbering of the nucleotides is relative to the ECH_0659 translational start codon. (C, D, E, and F) β-Gal activity for each construct is expressed as Miller units and was measured in Escherichia coli as described in Materials and Methods. The values are results from three independent experiments. Statistical significance was determined by one-way analysis of variance (ANOVA) followed by Tukey’s multiple-comparison test (**, *P < *0.01; ***, *P < *0.001; ****, *P < *0.0001).

Bioinformatic analysis of the putative 294-bp-long promoter segment that is located upstream of the coding sequence of the first of the seven genes revealed the presence of several inverted repeat (IR) sequences with the potential to form intrastrand secondary structures (Fig. 1). Furthermore, the sequence included putative binding sites that likely interact with zinc- and iron-responsive proteins (37). To test whether transcription from the seven genes could be influenced by these metal ions, the promoter segment was cloned in front of the *lacZ* reporter gene in a plasmid, and β-galactosidase activity was tested in E. coli XL1-Blue MRF′ under conditions of chemically defined medium adjusted by the availability of metal ions. To assess the zinc restriction, E. coli transformed with a plasmid vector (pJT3) was first grown under phosphate starvation conditions in the culture medium with or without 15 μM ZnSO_4_ for 24 h. The endogenous alkaline phosphatase (PhoA) activity expressed from the E. coli chromosome served as an ideal positive control for our study to measure the impact of zinc starvation assessed from the E. coli lysates, as it requires zinc for its optimal activity (38). A reduction of 40% of PhoA activity was observed without zinc, compared to when it was present (Fig. 2A). We then analyzed the activity for the E. coli lysate containing the recombinant plasmid with the full-length promoter (pJT294) or segments of it with 5′ end deletions (Fig. 2B). The β-galactosidase (β-Gal) activity was observed only for the recombinant plasmid containing the full-length promoter (Fig. 2C). Further, the β-Gal activity increased by 30% when zinc was absent in the culture medium compared to that when it was present (Fig. 2D). The 5′ end deletion segments of the promoter caused enhanced β-Gal expression when zinc was absent (Fig. 2E), with minimal β-Gal activity observed for the shortest segment of 50 bp. Similarly, the full-length recombinant construct expressed detectible β-Gal activity with or without 100 μM FeCl_2_ or FeCl_3,_ and the enzyme activity was significantly greater in the absence of iron compared to that in its presence (Fig. 2F).

### Transcripts from the seven genes assessed for the *E. chaffeensis* wild type and ECH_0660 and ECH_0665 mutants.

**FIG 3:**
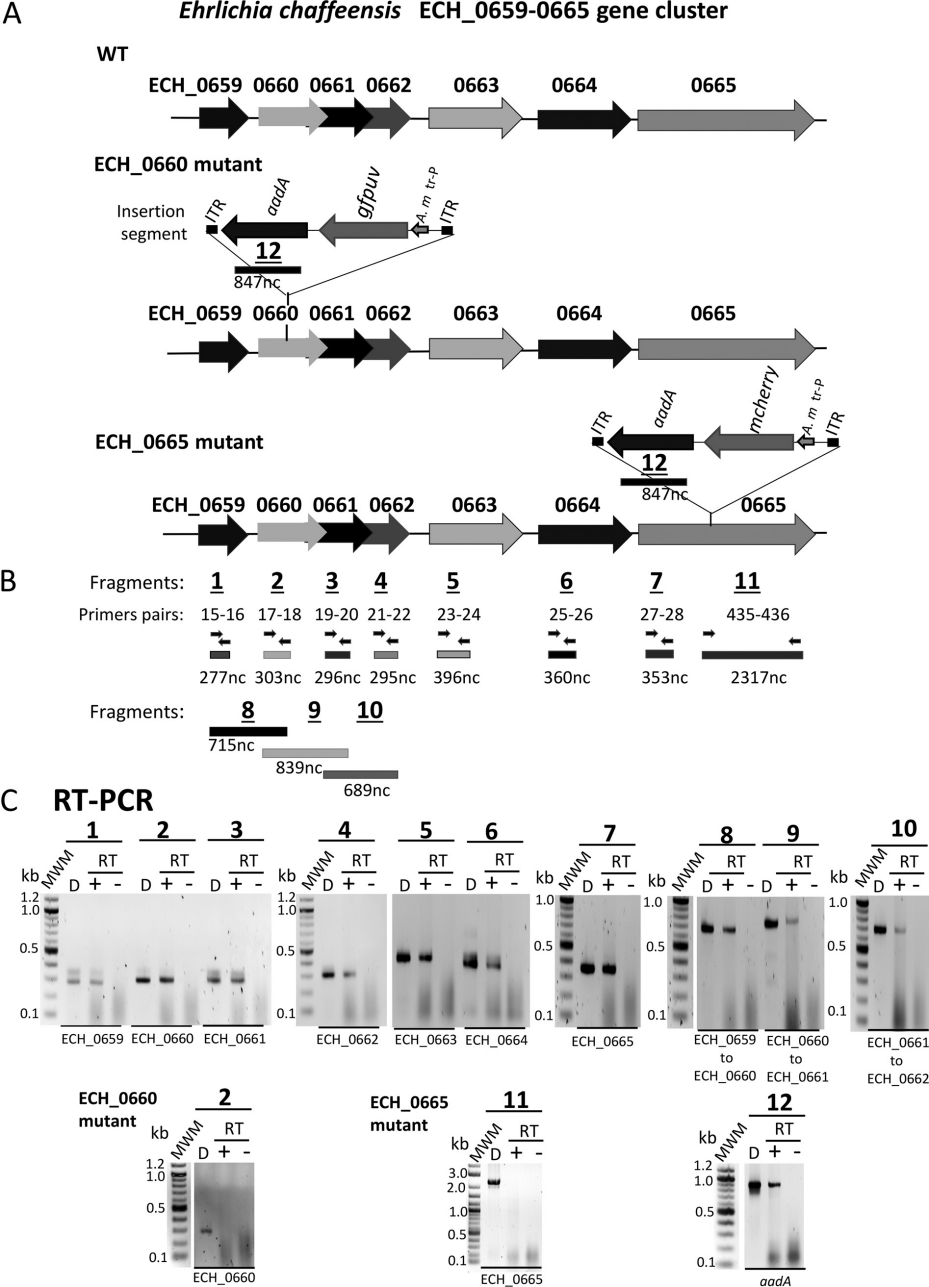
Transcripts from the seven genes assessed for the *E. chaffeensis* wild type and the ECH_0660 and ECH_0665 mutants. (A) Schematic representation of the seven genes on *E. chaffeensis* wild-type chromosome, and ECH_0660 and ECH_0665 mutants. (B) Reverse transcription-PCR (RT-PCR) targets identified. The primer pairs and their estimated products are represented with arrows and bars, respectively. (C) RT-PCR data presented for the amplicons generated targeting the 7 genes. “D” refers to a positive control with genomic DNA as the template; + and − refer to the RT-PCR assays performed with or without reverse transcriptase, respectively. Molecular weight markers (MWM) were included when resolving the PCR products to help locating specific amplicons. Expected amplicons for the internal coding regions of all seven genes were detected for the wild type when reverse transcriptase was added but were absent for the mutation insertion regions of ECH_0660 and ECH_0665 mutants (PCRs 2 and 11, respectively). Also, amplicons from overlapping coding regions of genes were detected as shown. For both ECH_0660 and ECH_0665 mutants, the transcription of the *aadA* gene from the Himar1 transposon (Tn) insertion was also observed (PCR 12).

Transcripts for all seven genes were detected in the RNA recovered from the *E. chaffeensis* wild type cultured in the canine macrophage cell line DH82 (Fig. 3A to C). As intergenic spacer regions are absent or too small and range in size from 43 to 141 bp between the genes of all seven genes (Fig. S1), and given that the promoter prediction analysis identified several putative binding sites for transcriptional regulators in the sequence upstream from the ECH_0659 coding region (Fig. 1), we assessed if a polycistronic message(s) is transcribed from these genes. Reverse transcription-PCR (RT-PCR) assays targeting the coding regions overlapping from ECH_0659 to ECH_0662 yielded predicted amplicons (Fig. 3B and C), while such products were absent when assessed for the presence of overlapping transcripts between the genes from ECH_0662 to ECH_0665 (data not shown). These results suggest that the genes ECH_0659 to ECH_0662 are cotranscribed as a single transcript, while the remaining three genes, spanning from ECH_0663 to ECH_0665, are not part of the polycistronic message. To determine the impact of the ECH_0660 gene disruption mutation (34), RT-PCR analysis was carried out similarly for this mutant. Transcription was not detected for ECH_0660 (Fig. 3C), although the mutant tested positive for the remaining six genes (data not shown). We recently generated another mutant with functional disruption in ECH_0665, the last gene of the gene cluster (39). Unlike the ECH_0660 mutation, this mutation has no impact on the pathogen’s *in vivo* growth, as it progressed very similarly to that of the wild type when assessed in a vertebrate host (39). For this mutant, transcription was absent for ECH_0665 (Fig. 3C), while the remaining gene transcripts were detected (data not shown). In both ECH_0660 and ECH_0665 mutants, the RT-PCR products targeting the insertion-specific *aadA* gene were also observed (Fig. 3C).

### Effects of zinc and iron starvation on the *E. chaffeensis* wild type and ECH_0660 and ECH_0665 mutant strains.

**FIG 4:**
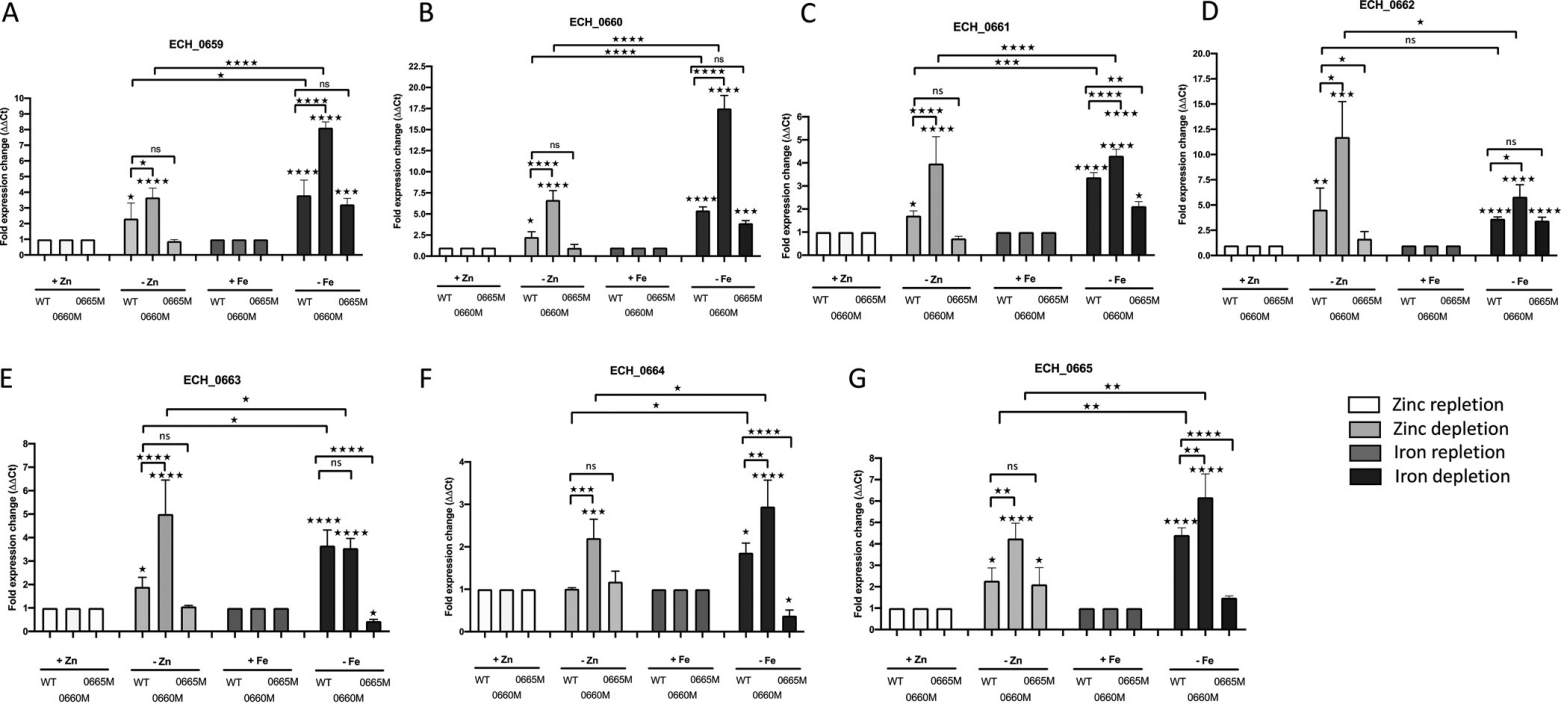
Effects of zinc and iron starvation on the *E. chaffeensis* wild type and ECH_0660 and ECH_0665 mutants. The genes ECH_0659 to ECH_0665 from *E. chaffeensis* are upregulated under zinc and iron depletion conditions. The expression of ECH_0659 (A), ECH_0660 (B), ECH_0661 (C), ECH_0662 (D), ECH_0663 (E), ECH_0664 (F), and ECH_0665 (G) from wild-type, ECH_0660 mutant, and ECH_0665 mutant organisms was measured during the stationary phase of infection under zinc repletion or depletion conditions, and similarly under iron repletion or depletion conditions, by quantitative real-time RT-PCR. The data represent the mean ± standard deviation (SD) from 2 biological replicas, each of which comprises 3 technical replicas. Fold expression changes are normalized using *gyrB* as an endogenous reference gene (Δ*C_T_*). Statistical significance was determined by one-way analysis of variance (ANOVA) followed by Tukey’s multiple-comparison test (*, *P* < 0.05; **, *P* < 0.01; ***, *P* < 0.001; ****, *P* < 0.0001).

As we found only the predicted promoter region upstream of the seven genes and because this promoter responded to zinc and iron depletion when assessed in the heterologous E. coli surrogate system, we evaluated how *E. chaffeensis* responds to zinc and iron starvation in culture. The *E. chaffeensis* wild type and two mutants with ECH_0660 or ECH_0665 gene function disruptions were assessed by reverse transcription-quantitative PCR (qRT-PCR) for transcriptional changes from the cluster of seven genes, following their growth in medium containing zinc and with or without the zinc chelator *N*,*N*,*N*′,*N*′-tetrakis(2-pyridylmethyl)ethylenediamine (TPEN) (Fig. 4). We similarly assessed the iron starvation in medium containing FeSO_4_ and with or without the inclusion of the iron chelator 2,2′-bipyridyl (BIP) (Fig. 4). The relative gene expression in the presence of the zinc chelator for ECH_0659 was increased 2.3-fold for the wild type and 3.7-fold for the ECH_0660 mutant but did not show significant alteration in ECH_0665 mutant (Fig. 4A). The iron chelation is more pronounced for this gene, where we observed a 3.8-fold increase for the wild type. Similarly, for the ECH_0665 mutant, the transcripts increased by 3.2-fold, whereas an 8.1-fold increase was observed for the ECH_0660 mutant (Fig. 4A). Similarly, ECH_0660 transcripts for zinc chelation resulted in a 2.3-fold increase for the wild type and a 6.7-fold increase for the ECH_0660 mutant for gene coding region upstream of the insertion mutation site, while 1-fold downregulation was noted for the ECH_0665 mutant (Fig. 4B). The iron chelation was also more enhanced for these gene transcripts, which increased by 5.4-fold for the wild type and 17.5-fold for the ECH_0660 mutant, whereas it declined 1.5-fold for the ECH_0665 mutant compared to that of the wild type (Fig. 4B). For ECH_0661, the relative transcription without zinc was increased 1.7-fold for the wild type, while expression increased 4-fold for the ECH_0660 mutant and decreased to 0.7-fold for the ECH_0665 mutant (Fig. 4C). As in ECH_0659 and ECH_0660 gene transcripts, iron chelation resulted in considerably more transcripts for ECH_0661; these gene transcripts increased to 3.4-fold, 4.3-fold, and 2.1-fold for the wild type, ECH_0660 mutant, and ECH_0665 mutants, respectively (Fig. 4C). The removal of zinc from the medium caused a much more pronounced enhancement of the ECH_0662 transcription, as 4.6-fold and 11.7-fold transcript levels were observed in the wild type and the ECH_0660 mutant, respectively, and with no significant difference for the ECH_0665 mutant (Fig. 4D). While iron chelation also triggered ECH_0662 transcription, its effect is less compared to that of zinc chelation, with similar values for the wild type and ECH_0665 mutants (3.6- and 3.5-fold, respectively), whereas for the ECH_0660 mutant, it was another 2-fold more (5.8-fold) (Fig. 4D). ECH_0663 transcript levels increased during zinc chelation 1.9-fold for the wild type and 5-fold for the ECH_0660 mutant, with no significant change for the ECH_0665 mutant, whereas for the iron chelation, the wild type and the ECH_0660 mutant had similar increases of transcripts (3.7- and 3.6-fold, respectively), and transcription declined 0.5-fold for the ECH_0665 mutant (Fig. 4E). ECH_0664 relative gene expression was moderately increased during zinc chelation only for the ECH_0660 mutant bacteria, while no differences in the transcript levels were observed for both the wild type and the ECH_0665 mutant (Fig. 4F). Similarly, a moderate increase in transcript levels was observed in bacteria cultured under conditions of iron chelation for the wild type (1.9-fold) and the ECH_0660 mutant (3-fold) but was downregulated for the ECH_0665 mutant (0.4-fold) (Fig. 4F). Gene expression changes for the last gene (ECH_0665) also increased moderately, about 2.3-fold for the wild type and 2.1-fold for the ECH_0665 mutant (for the gene coding region upstream of the mutation site), and 4.3-fold for the ECH_0660 mutant. With the iron chelation, the transcription enhancement was more noticeable; 4.4-fold and 6.2-fold, respectively, for the wild type and ECH_0660 mutant bacteria and no significant difference for the ECH_0665 mutant (Fig. 4G). Together, transcripts of all seven genes were significantly upregulated for the wild type under both the zinc and iron depletion conditions, and the transcription levels were significantly enhanced for the ECH_0660 mutant, while essentially similar enhancements were observed in the wild type and downregulation was observed in the ECH_0665 mutant.

## DISCUSSION

In earlier studies, we reported that a mutation in the *E. chaffeensis* ECH_0660 gene encoding the phage head-to-tail connector protein results in the rapid clearance of the pathogen from vertebrate hosts (white-tailed deer and dogs). In contrast, persistent infection occurs with wild-type *E. chaffeensis* and the ECH_0665 mutant (34, 35, 39, 40). Pathogenic intracellular bacteria are often challenged by the restricted availability of metal ions such as iron and zinc in the host cells (20). Starvation of the metal ions triggers enhances expression of certain metal ion response genes in bacteria. In this study, we characterized functional disruption mutations in the ECH_0660 and ECH_0665 genes of *E. chaffeensis* encoding two predicted phage proteins, the head-to-tail-connector protein and the phage terminase enzyme, respectively. We assessed how the wild type and the two mutants respond to depletion of two important metal ions, zinc and iron. The wild type and the mutant bacteria had distinct gene expression profiles in response to depletion of the two metal ions. Specifically, chelation of the metal ions triggered significant transcriptional enhancement for most of the genes from the seven genes for the wild type. Transcription enhancement was significantly higher for all seven genes under zinc starvation conditions for the ECH_0660 mutant, while iron starvation similarly caused enhanced gene expression from six genes. Contrary to these observations, the transcripts for the ECH_0665 gene mutant, previously shown to have a minimal impact on the bacterial *in vivo* growth (39), were more or less similar to those of wild-type *E. chaffeensis*. We used TPEN, a chelator known to chelate other divalent metal ions such as cadmium, cobalt, nickel, and copper (41), to chelate zinc from *E. chaffeensis* cultures. Thus, the gene expression changes observed may also be the result of depleting other metal ions. As the E. coli surrogate system selectively demonstrated the impact of zinc, we reasoned that the TPEN chelation effects primarily resulted from zinc starvation.

Zinc and iron play essential roles in bacterial cells to serve as cofactors for multiple enzymes, to support the structural integrity of proteins, and to regulate protein expression from several bacterial genes (42). These metal ions are not readily accessible for intracellular pathogens (20). Pathogenic bacteria employ unique strategies in acquiring metal ions from a host cell during intracellular replication (24). We observed that the predicted promoter sequence upstream of the seven genes activates reporter gene expression, and it was significantly higher in medium with limited zinc or iron when assessed in the E. coli surrogate system. Although the E. coli*-*based analysis may not be ideal for assessing an *E. chaffeensis* promoter function, this heterologous system is currently the best means of investigating bacterial gene regulation, considering the lack of desirable genetic tools for the pathogen (11, 43–49). Assessment of the effect of promoter deletion analysis suggested that one or more of the putative DNA binding motifs within the sequence responds to zinc or iron depletion. The *E. chaffeensis* genome includes genes encoding a zinc transporter protein (ECH_0067) and an iron transport system substrate-binding protein (ECH_0189). However, it is yet to be determined if these proteins are involved in metal ion transport. A recent study in a related Ehrlichia species (Ehrlichia ruminantium) suggested that iron restriction causes upregulation of a T4SS protein (VirB), an outer membrane-associated protein (MAP1), and two predicted transcriptional regulators (Tr1 and ErxR) (28). In the current study, we demonstrated significant transcriptional enhancement for the seven genes when depleting zinc and iron during *E. chaffeensis* growth for the wild-type bacteria. The transcriptional enhancement was significantly greater for the ECH_0660 mutant, a mutation known to exert an *in vivo* growth defect (34, 35, 50). In contrast, transcript levels were similar to those of the wild type, or slightly downregulated for the ECH_0665 mutant. We recently reported that this mutation has a minimal impact on its *in vivo* growth (39). Together, the data presented in the current study suggest that the growth defect mutation in the ECH_0660 gene encoding the phage head-to-tail connector protein triggers significant upregulation of transcription from the seven genes assessed when metal ions are chelated, while the mutation in the ECH_0665 gene encoding the phage terminase protein leads to the pathogen maintaining transcription levels more or less similar to those of wild-type *E. chaffeensis*.

We reported earlier that the ECH_0660, ECH_0663, and ECH_0665 gene products are membrane-associated proteins, as judged by mass spectrometry analysis of purified total and immunogenic *E. chaffeensis* membrane fractions (51). We recently reported that the ECH_0660 gene mutant caused minimal variation in the bacterial global transcriptome and proteomes compared to the wild-type *E*. *chaffeensis* (52, 53) when assessed in standard culture medium (54). This mutant having the growth defect allowed the vertebrate host to initiate a potent immune response to confer protection against wild-type pathogen infection challenge (34, 35). The data presented in the current study suggest that the functional disruption in the predicted phage head-to-tail connector protein gene is likely detrimental in altering the pathogen’s ability to obtain metal ions to support its growth within a phagosome of infected host macrophages. Our previously described mutation in the ECH_0660 gene causing *in vivo* attenuation may have caused a metabolic burden for *E. chaffeensis* resulting in the upregulation of gene expression from several genes likely involved in the metal ion uptake.

## MATERIALS AND METHODS

### Bioinformatics.

The *E. chaffeensis* genomic region (GenBank accession number CP000236.1) spanning from the ECH_0659 to ECH_0665 genes was subjected to data searching as described below. Inverted repeat and direct repeat sequences in DNA were identified by visual examination and by using the palindromic sequence finder REPFIND (55). Promoter −35 and −10 element sequences for *Ehrlichia* genes were predicted with the BPROM program (56). Genomic DNA sequences of Ehrlichia canis (Jake), Ehrlichia muris (AS145), Ehrlichia ruminantium (Welgevonden), Anaplasma phagocytophilum (HZ), Anaplasma marginale (St. Maries), Wolbachia (wHa), and Neorickettsia sennetsu were obtained from the NCBI website. The genome data were compared with the gene cluster from ECH_0659 to ECH_0665 to identify the gene homologs. DNA and protein alignments were performed with ClustalW and ClustalX, respectively. Database searches were performed using NCBI BLAST and CDD databases (57).

### Bacterial strains, plasmids, and media.

The bacterial strains and plasmids used in this study are listed in Table S1 in the supplemental material. Escherichia coli strains were routinely grown at 37°C in Luria–Bertani (LB) medium. When required, the medium was supplemented with an appropriate antibiotic. Tris-glucose (TG) medium (58) was used for zinc response assessment, with or without 15 μM ZnSO_4_. Similarly, the iron response was assessed in TG medium with or without 100 μM FeCl_2_ or 100 μM FeCl_3_. For determining β-galactosidase (β-Gal) or alkaline phosphatase (PhoA) activities, 100-μl aliquots taken at 24 h or 72 h were used as described below.

### Construction of ECH_0659 promoter-*lacZ* fusion plasmids.

DNA manipulations to prepare recombinant plasmids and E. coli transformations were carried out by standard molecular cloning protocols as in (59). All primers used in this study were synthesized by Integrated DNA Technology (Coralville, IA) and are listed in Table S2 in the supplemental material. DNA fragments containing the entire 294-bp putative promoter region upstream of the ECH_0659 gene coding sequence were amplified by PCR using *E. chaffeensis* genomic DNA as the template and with the primer sets specific for the segment (Table S2). PCR products were cloned using primers designed for directional cloning into KpnI-BamHI sites of the pJT3 plasmid (60) to create the recombinant plasmid pJT294. Deletion constructs were prepared similarly with 5′-end sequential deletions.

### Enzymatic assays.

β-Galactosidase (β-Gal) activity was assessed on the cultures supplemented with 50 μg 5-bromo-4-chloro-3-indolyl-β-d-galactopyranoside (X-Gal) · ml^−1^. Quantitative evaluation of β-Gal was carried out using permeabilized cells obtained from stationary-phase cultures grown with or without 15 μM ZnSO_4_, or with or without 100 μM FeCl_2_/FeCl_3_, and incubated with *o*-nitrophenyl-β-d-galactopyranoside (ONPG) substrate (Sigma-Aldrich) as previously described (61). Quantitative evaluation of alkaline phosphatase (PhoA) was carried out similarly using cultures grown under phosphate starvation conditions with or without 15 μM ZnSO_4_ and incubated with 4-nitrophenyl phosphate (4 mg · ml^−1^) substrate (Sigma-Aldrich) as described previously (58). Average values (±standard deviations) for activity units were calculated from three independent assays, each carried out in triplicate.

### Treatment of *E. chaffeensis*-infected macrophages (DH82 cells) with metal chelators and subsequent RNA analysis.

*E. chaffeensis* (Arkansas isolate) wild-type, ECH_0660 mutant (34), and ECH_0665 mutant (39) cells were grown in the canine macrophage cell line DH82 (ATCC, Manassas, VA) in minimal essential medium (MEM) as previously described (54). For the zinc response assay, culture medium was supplemented with 15 μM ZnSO_4_, with or without 5 μM *N*,*N*,*N*′,*N*′-tetrakis(2-pyridylmethyl)ethylenediamine (TPEN, a zinc chelator; Sigma-Aldrich, St. Louis, MO) (63). To define the growth characteristics under iron-supplemented or -depleted conditions, *E. chaffeensis* cultures were propagated in medium supplemented with 100 μM FeSO_4_ and with or without 0.15 mM 2,2′-bipyridyl (BIP, an iron chelator; Sigma-Aldrich) (62). TPEN or BIP was added independently to the cultures at 72 h postinoculation when infectivity was about 80 to 90%. After 24 h, cultures were harvested by centrifugation at 14,000 × *g* for 10 min. The pellet was resuspended with a half volume of the medium and mixed with 2.5 ml of RNAlater stabilization solution (Thermo Fisher) and stored at −4°C until RNA extraction. Total RNA was recovered, DNase treated, and then reverse transcribed using SuperScript III reverse transcriptase (Invitrogen). The cDNA was used in PCR analysis using Platinum *Taq* DNA polymerase (Invitrogen) using primers specific for each gene. The resulting PCR products were separated by electrophoresis in 1.5% agarose gel. Real-time PCR amplification of the cDNA was performed with 2× PowerUp SYBR green master mix (Applied Biosystems, Life Technologies) as specified by the manufacturer, with 1 μl cDNA (corresponding to 0.1 to 0.2 μg of total RNA) and using a gene-specific primer set. Two independent experiments were performed for every gene transcript with samples examined in triplicate for each experiment in a StepOnePlus real-time PCR system (Applied Biosystems by Life Technologies). Relative expression levels of the specific transcripts between different biological conditions for wild-type and mutant *E. chaffeensis* were calculated using the relative abundance of *gyrB* as an internal reference standard for normalization and expressed as fold differences by calculating 2^−(ΔΔ^*^CT^*^)^ (*C_T_*, threshold cycle) (64). The final qRT-PCR data were presented as the means of two experiments.

### Statistics.

The statistical differences between experimental groups were assessed by one-way analysis of variance (ANOVA) followed by Tukey’s multiple-comparison test or Student’s *t* test. Differences with a *P* value of <0.05 were considered significant.
