# Supplementary material for: Mutations in Ehrlichia chaffeensis Genes ECH_0660 and ECH_0665 Cause Transcriptional Changes in Response to Zinc or Iron Limitation
Source: J Bacteriol. 2021 Jun 8;203(13):e00027-21. doi: 10.1128/JB.00027-21 (PMC8316085; doi:10.1128/JB.00027-21)
Supplement: Supplemental file 1 — Tables S1 and S2 and Fig. S1 to S3. Download JB.00027-21-s0001.pdf, PDF file, 3.41 MB [file jb.00027-21-s0001.pdf]

Table S1: strains and plasmids used in this work

Strain and plasmid	Derived, relevant genotype, or characteristic	Source or reference
Escherichia coli		
XL1-Blue MRF’	□(*mcrA*)*183* □(*mcrCB-hsdSMR-mrr*)*173* *endA*1 *supE*44 *thi*-1 *recA*1 *gyrA*96 *relA*1 *lac* (F’ *proAB* $lacI^q$ *Z*□M15 Tn*10* (Tet^r).	Stratagene
Ehrlichia strains		
E. chaffeensis Arkansas wild-type		Laboratory stock
ECH_0660 mutant	Arkansas, *E. chaffeensis* ECH_0660::*Himar1* Tn	Cheng *et al.,* 2013
ECH_0665 mutant	Arkansas, *E. chaffeensis* ECH_0665::*Himar1* Tn	Wang *et al.* 2020
Plasmids		
pJT3	Km^r, promoterless *lacZ* reporter vector	Juárez-Rodríguez *et al.* 2013
Derived from reporter vector pJT3		
pJT294	Km^r, pJT3, $P_{ECH\_0660\ (294\text{-}bp)}$-(ATG)ECH_0660-*lacZ*	This study
pJT189	Km^r, pJT3, $P_{ECH\_0659\ (189)}$-(ATG)ECH_0659-*lacZ*	This study
pJT145	Km^r, pJT3, $P_{ECH\_0659\ (145)}$-(ATG)ECH_0659-*lacZ*	This study
pJT130	Km^r, pJT3, $P_{ECH\_0659\ (130)}$-(ATG)ECH_0659-*lacZ*	This study
pJT96	Km^r, pJT3, $P_{ECH\_0659\ (96)}$-(ATG)ECH_0659-*lacZ*	This study
pJT50	Km^r, pJT3, $P_{ECH\_0659\ (50)}$-(ATG)ECH_0659-*lacZ*	This study

Kanamycin resistance Km^r

Table S2. Oligonucleotides used in this work.
Related products and oligonucleotides   Sequence (5'→3')

Fusion *lacZ* promoterless (single)

ECH_0659
294 bp
53z-F CAGCCTCTCGGTACCATCTCTCCACCTTACACTTC
9z-R TCATCTAGTTTTTTTTGTTGGGATCCCATAAAAGTTTGTTTGCTGTGG

Delimitation of the Inter genetic region (IGR)-294 bp

189 bp
54z-F GCAAATTTCAATAAAATTCGGTACCTTTACTATCAACGTTCAAC
9z-R TCATCTAGTTTTTTTTGTTGGGATCCCATAAAAGTTTGTTTGCTGTGG

145 bp
62z-F GAATGTGCAGTTAACGAATTCGAGCTCTACTTTTTTAAATAGTAATACAAAACCTTCCAATG
46v-R CCTGCAGGTCGACTCTAGATCATGAGC

45v-F GCTCATGATCTAGAGTCGACCTGCAGG
63z-R CATTGGAAGGTTTTGTATTACTATTTAAAAAAGTAGAGCTCGAATTCGTTAACTGCACATTC

130 bp
67z-F GAATGTGCAGTTAACGAATTCGAGCTCTAATACAAAACCTTCCAATGAAAACATAAATAAACTGC
46v-R CCTGCAGGTCGACTCTAGATCATGAGC

45v-F GCTCATGATCTAGAGTCGACCTGCAGG
68z-R GCAGTTTATTTATGTTTTCATTGGAAGGTTTTGTATTAGAGCTCGAATTCGTTAACTGCACATTC

96 bp
55z-F CCTTCCAATGAAAACATAAATAAGGTACCTGCTTCCTCAATACC
9z-R TCATCTAGTTTTTTTTGTTGGGATCCCATAAAAGTTTGTTTGCTGTGG

50 bp
69z-F GAATGTGCAGTTAACGAATTCGAGCTCATATTAATATTACTATGACAAAAACTTCTCACCACAG
46v-R CCTGCAGGTCGACTCTAGATCATGAGC

45v-F GCTCATGATCTAGAGTCGACCTGCAGG
70z-R CTGTGGTGAGAAGTTTTTGTCATAGTAATATTAATATGAGCTCGAATTCGTTAACTGCACATTC

RT-PCR primers

ECH_0659
15f CTAGATGAATTTGACTATACAATTGATGATATTATTAC
16r CACTAAAAGAAATTTTGTTAAATATTTTAGTGATGATG

ECH_0660
17f CAGAAGTAAAATCCTTCCTACGCATTAAC
18r CCATGGATATGCTTATTAAATGAAATGTATGATAG

ECH_0661
19f CAATTTAACTAATACTGTAACTGATATATACGACATC
20r GTGCATATGCAATTTCATTGTGCTGATTTG
ECH_0662
21f CTTACGAATTAGCTTACATAACAAAAACGAATAC
22r GCTTATTAAAACCATATTGAAATTGTCAAAATTACTTG

ECH_0663

23f	CCACAATAAAACAAGTATCTTCGAAAGAATAC
24r	GATTTTCAATTTTCTGATTTTCATCTTTACTATTTGTAG

ECH_0664

25f	GCAATATACCATCATATTGATATATCTGATAATATTAATG
26r	CATGCTTTTTGATTTGTGTGTATGGCTAG

ECH_0665

27f	GCATCTCAAAATCATAACAGACAGACTG
28r	CACCTTCTCCTGTAATACTTCCTCC

ECH_0665 Mutant

435	CTCTGTTACTTCACCATGGAAATTTAAATATG
436	CTTAATTCTTGAATAATTTGTAATCCACTTGC

aadA1

415f	CACAGTGATATTGATTTGCTGGTTAC
416r	GTGCACAACAATGGTGACTTCTAC

qRT-PCR primers

ECH_0659

38f	CATTTTCAACACTCTCTAGAAGAGG
39r	GTGATGATGTCTTTCTCATATTTATTTAAGC

ECH_0660 (For amplification of the region upstream of the Tn Himar1 interruption)

40f	CTTATCAGAAGTAAAATCCTTCCTACG
41r	AGGAAATTTCCCATGTTTGTTTCATTAATG

ECH_0661

42f	CGACATCATACCTAATAAAGTATCATTAC
43r	GTGTATTGGGTTGGTAGCAGTAG

ECH_0662

44f	CGAATTAGCTTACATAACAAAAACGAATAC
45r	TGTAAAAGTTGATTTGCAGAGGAATAATC

ECH_0663

46f	CTGCTCTATTAAAAATAACACAGGATAAATC
47r	CTAATATTATTCTCTAGTTCTGCATTTTCTTC

ECH_0664

48f	GACTCTATTAATGTCAACCATAGCTC
49r	GCTTTTTGATTTGTGTGTATGGCTAG

ECH_0665 (For amplification of the region upstream of the Tn Himar1 interruption)

49f	GCTTTTTGATTTGTGTGTATGGCTAG
50r	CAATCGTTTAATCATCAACATGCCTC

ECH_0620 gyrB

323f	CTGTTTCTGTTGTGGATAATGGTAG
324r	CACCATGAAGTCCACCTGAAAC

Fig. S1

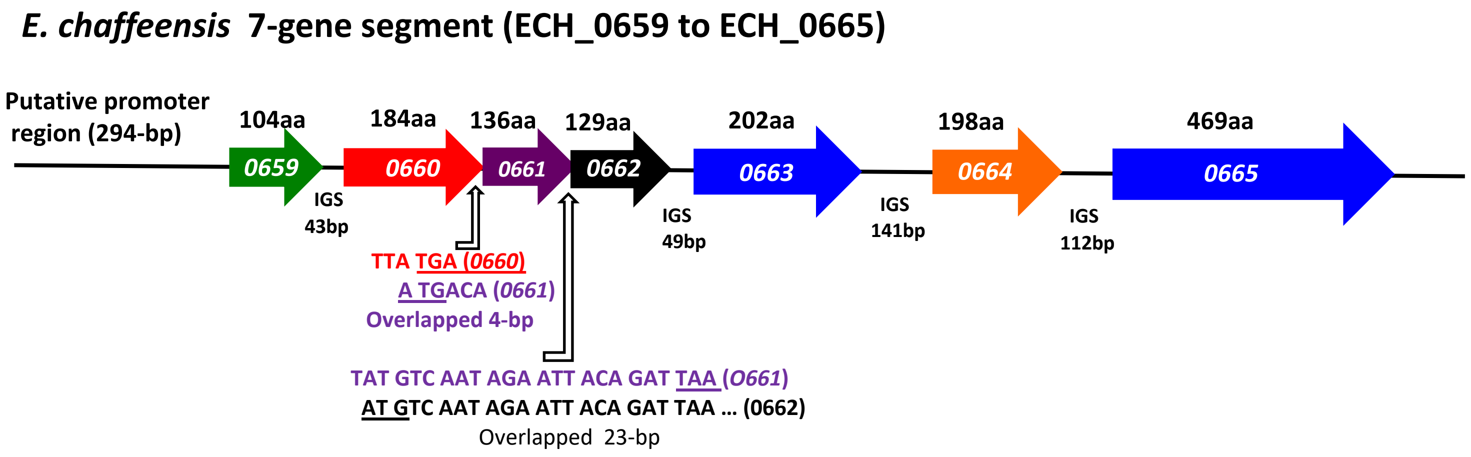

Figure S1. Genomic organization of the 7-gene cluster spanning ECH_0659 to ECH_0665.
Cartoon depicting genomic region representing the 7-gene cluster spanning from ECH_0659 to ECH_0665 genes. Putative promoter region (294 bp) upstream to the first gene of the segment (ECH_0659) is identified. Gene transcription orientation is indicated by open arrows. Intergenic spacer (IGS) regions and the length of sequences where applicable are identified. When an open reading is overlapped between two adjacent genes, the overlapping sequences are identified in the Figure. The predicted protein size in number of amino acids (aa) for gene coding regions is shown above each gene.

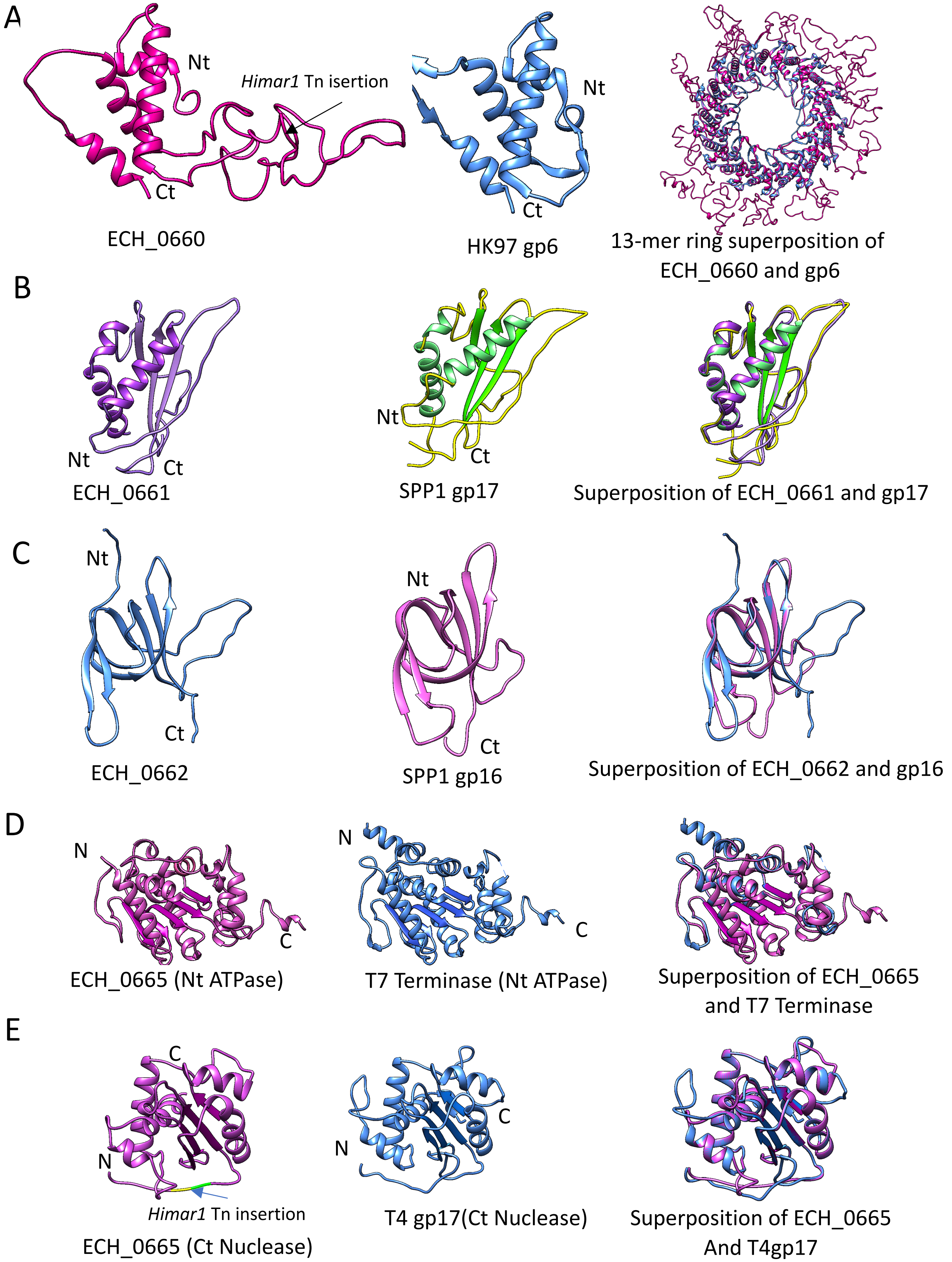
A
Nt
Himar1 Tn isertion
Ct
ECH_0660
Nt
Ct
HK97 gp6
13-mer ring superposition of ECH_0660 and gp6
B
Nt
Ct
ECH_0661
Nt
Ct
SPP1 gp17
Superposition of ECH_0661 and gp17
C
Nt
Ct
ECH_0662
Nt
Ct
SPP1 gp16
Superposition of ECH_0662 and gp16
D
N
C
ECH_0665 (Nt ATPase)
N
C
T7 Terminase (Nt ATPase)
Superposition of ECH_0665 and T7 Terminase
E
C
N
Himar1 Tn insertion
ECH_0665 (Ct Nuclease)
N
C
T4 gp17(Ct Nuclease)
Superposition of ECH_0665 And T4gp17

Fig. S2. Prediction analysis of proteins encoded by ECH_0660, ECH_0661, ECH_0662 and ECH_0665 genes, respectively. Three-dimensional models of: (A) ECH_0660 and compared with HK97 gp6 protein, and superposition of 13-mer ring structure of ECH_0660 and HK97 gp6; (B) ECH_0661 compared with SSP1 gp17 protein, and superposition of ECH_0661 and SSP1 gp17; (C) ECH_0662 compared with SSP1 gp16 protein, and superposition of ECH_0662 and SSP1 gp16; (D) ECH_0665 N-terminal ATPase domain compared with N-terminal T7 large terminase; and superposition of both proteins; (E) ECH_0665 C-terminal Nuclease domain compared with C-terminal Nuclease T4 gp17, and superposition of both proteins. Models were constructed with Swiss Model (Guex *et al.* 2009) and Chimera (Meng *et al.* 2006) software. The templates used to build the models were: PDB id:3jvo for Ech_0660, PDB id:2flp for ECH_0661, PDB id:2kca for ECH_0662, PDB id: 4bijd for ECH_0665 ATPase domain and PDB id: 3ezkb for ECH_0665 Nuclease domain.

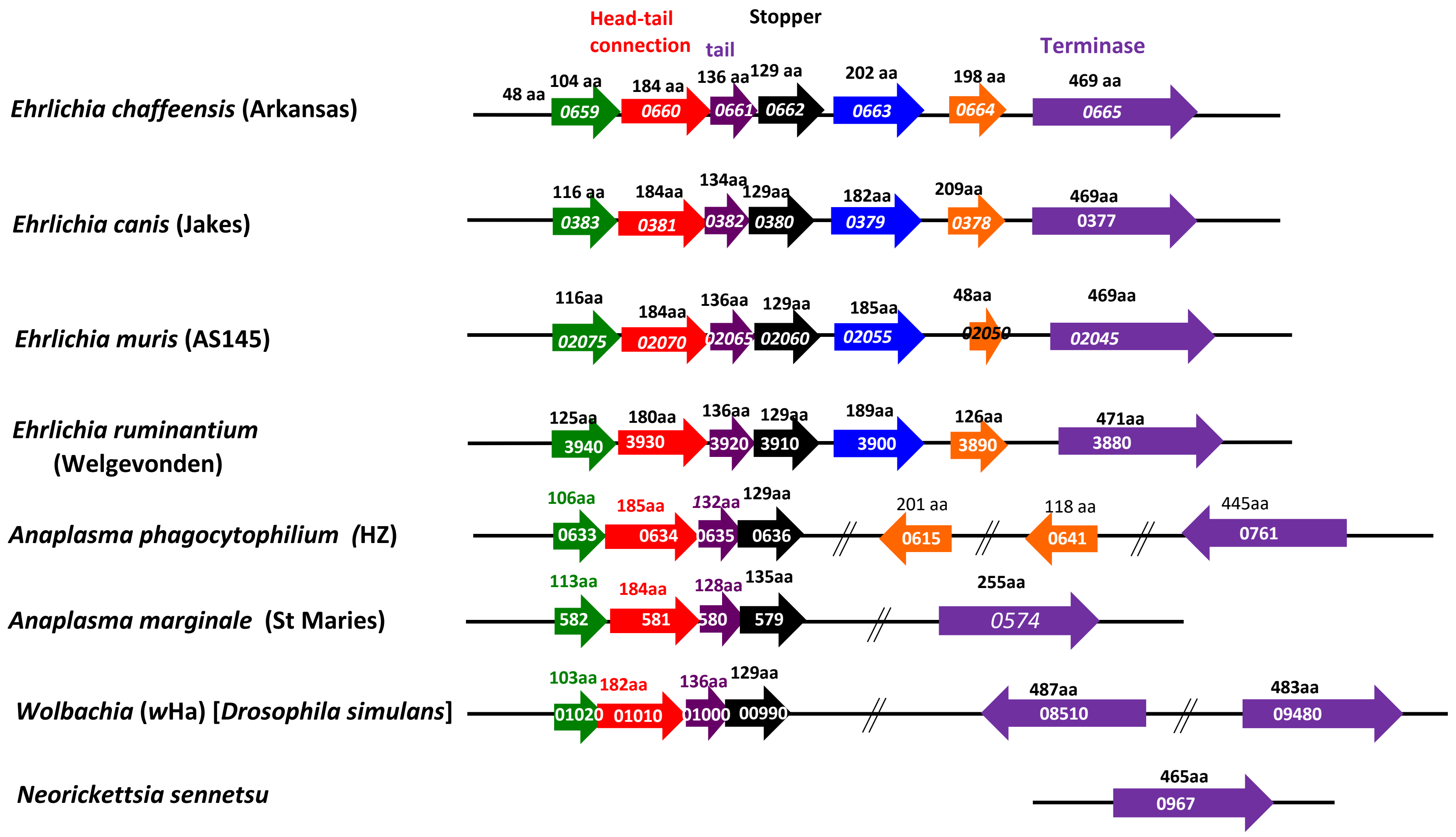

Fig S3. The 7-gene cluster conservation assessed in related *Anaplasmataceae* family bacteria. Genomic organization and the existence of the homologs of the 7-gene cluster of *E. chaffeensis* in several related rickettsials belong to the *Anaplasmataceae* family were assessed by searching genome data from the organisms listed in the Figure for the presence of *E.* chaffeensis 7-gene cluster homologs. The data are presented in cartoon form when one or more gene homologs are found. Orientation of gene coding regions are identified with the direction of the arrowheads.

REFERENCES:

Cheng C, Nair ADS, Indukuri VV, Gong S, Felsheim RF, et al. (2013) Targeted and Random Mutagenesis of *Ehrlichia chaffeensis* for the Identification of Genes Required for In vivo Infection. PLoS Pathog 9(2): e1003171. https://doi:10.1371/journal.ppat.1003171

Wang Y, Nair ADS, Alhassan A, Jaworski DC, Liu H, Trinkl K, Hove P, Ganta CK, Burkhardt N, Munderloh UG, Ganta RR. 2020. Multiple Ehrlichia chaffeensis genes critical for its persistent infection in a vertebrate host are identified by random mutagenesis coupled with in vivo infection assessment. Infect Immun 88:e00316-20. https://doi.org/10.1128/IAI.00316-20.

Juárez-Rodríguez MD, Torres-Escobar A, Demuth DR. 2013. Construction of new cloning, *lacZ* reporter and scarless-markerless suicide vectors for genetic studies in *Aggregatibacter actinomycetemcomitans*. Plasmid, 69(3), 211–222. https://doi.org/10.1016/j.plasmid.2013.01.002

Guex N, Peitsch MC, Schwede T. 2009. Automated comparative protein structure modeling with SWISS-Model and Swiss-PdbViewer; a historical perspective. Electrophoresis. Suppl1:S162-73. https://doi.org/10.1002/elps.200900140

Meng EC, Pettersen EF, Couch GS, Huang CC, Ferrin TE. 2006.Tools for integrated sequence structure analysis with UCSF Chimera. BMC Bioinformatics.7:339. https://doi.org/10.1186/1471-2105-7-339
